# Exploring the implications of blocking renin-angiotensin-aldosterone system and fibroblast growth factor 23 in early left ventricular hypertrophy without chronic kidney disease

**DOI:** 10.3389/fendo.2023.1276664

**Published:** 2023-12-19

**Authors:** Kentaro Watanabe, Hideki Fujii, Kohei Okamoto, Keiji Kono, Shunsuke Goto, Shinichi Nishi

**Affiliations:** Division of Nephrology and Kidney Center, Kobe University Graduate School of Medicine, Kobe, Japan

**Keywords:** fibroblast growth factor 23, renin-angiotensin-aldosterone system, left ventricular hypertrophy (LVH), aldosterone (Ald), angiotensin-converting enzyme 2 (ACE2)

## Abstract

**Background:**

Whether fibroblast growth factor 23 (FGF23) directly induces left ventricular hypertrophy (LVH) remains controversial. Recent studies showed an association between FGF23 and the renin-angiotensin-aldosterone system (RAAS). The aim of this study was to investigate changes in FGF23 levels and RAAS parameters and their influences on LVH.

**Methods:**

In the first experiment, male C57BL/6J mice were divided into sham and transverse aortic constriction (TAC) groups. The TAC group underwent TAC at 8 weeks of age. At 1, 2, 3, and 4 weeks after TAC, the mice were sacrificed, and blood and urine samples were obtained. Cardiac expressions of FGF23 and RAAS-related factors were evaluated, and cardiac histological analyses were performed. In the second experiment, the sham and TAC groups were treated with vehicle, angiotensin-converting enzyme (ACE) inhibitor, or FGF receptor 4 (FGFR4) inhibitor and then evaluated in the same way as in the first experiment.

**Results:**

In the early stage of LVH without chronic kidney disease, serum FGF23 levels did not change but cardiac FGF23 expression significantly increased along with LVH progression. Moreover, serum aldosterone and cardiac ACE levels were significantly elevated, and cardiac ACE2 levels were significantly decreased. ACE inhibitor did not change serum FGF23 levels but significantly decreased cardiac FGF23 levels with improvements in LVH and RAAS-related factors, while FGFR4 inhibitor did not change the values.

**Conclusions:**

Not serum FGF23 but cardiac FGF23 levels and RAAS parameters significantly changed in the early stage of LVH without chronic kidney disease. RAAS blockade might be more crucial than FGF23 blockade for preventing LVH progression in this condition.

## Introduction

Cardiovascular disease (CVD) is a serious problem affecting the prognosis of patients with chronic kidney disease (CKD) (1). left ventricular hypertrophy (LVH) is the most common cardiac abnormality and is an independent risk factor for CVD mortality in patients with CKD (2, 3). Many risk factors, such as hemodynamic overload, uremic toxins, anemia, and activation of the renin-angiotensin-aldosterone system (RAAS), are involved in LVH in patients with CKD (4, 5). In addition to these factors, CKD-mineral bone disorder (CKD-MBD) plays an important role in LVH progression in patients with CKD. Although fibroblast growth factor 23 (FGF23) is a phosphaturic hormone, recent experimental and clinical studies have reported that FGF23 is related to CVD progression, and CVD and all-cause mortality (6). Serum FGF23 levels have been reported to be highly elevated particularly at the advanced stage of CKD (7).

Previous clinical and experimental studies showed that serum FGF23 levels were significantly associated with LVH in patients with CKD (8–12), whereas other previous clinical and experimental studies revealed that there was no significant relationship between FGF23 and LVH (13–15). Thus, the influence of FGF23 on LVH progression remains controversial (16). In addition, several recent studies showed an association between FGF23 and RAAS in LVH (17). The detailed changes in FGF23 levels and RAAS parameters, and their relationships during LVH progression remain unclear. In the present study, we firstly investigated the changes in serum and intracardiac FGF23 levels and RAAS parameters in the early stage of LVH progression, and secondly assessed the influences of FGF23 and RAAS blockade on their changes and LVH, using a pressure-overload induced LVH model mouse without CKD.

## Materials and methods

### Animals

Male C57BL/6 mice were obtained from Japan SLC, Inc. (Shizuoka, Japan). The mice were housed under light- and temperature-controlled environments and were fed a standard diet with water available ad libitum.

We created a pressure-overload-induced LVH model by transverse aortic constriction (TAC), using male C57BL/6 mice. At 8 weeks of age, the mice underwent TAC or sham operation. They were anesthetized with a combination of 0.3 mg/kg of medetomidine (Domitor; Nippon Zenyaku Kogyo Co., Ltd., Fukushima, Japan), 4.0 mg/kg of midazolam (Dormicum; Astellas Pharma Inc., Tokyo, Japan), and 5.0 mg/kg of butorphanol (Vetorphale; Meiji Seika Pharma, Ltd., Tokyo, Japan) via intraperitoneal administration and then intubated and attached to an experimental animal ventilator (MK-AT210D; Muromachi Kikai Co., Ltd., Tokyo, Japan). The ventilator settings were as follows: breathing rate, 120 breaths per minute; tidal volume, 0.3 mL. After disinfection of the skin with 70% alcohol and thoracic incision, through the second intercostal space, the thymus was separated and the transverse aorta was exposed. A 5-0 silk suture was tied around the transverse aorta with a 27-gauge needle (Terumo Co., Ltd., Tokyo, Japan) to produce a constriction after the removal of the needle. The muscles and skin were closed, and after spontaneous breathing appeared, the mice were extubated and moved into a cage placed on a pad that was maintained at 37°C. In the sham group, the surgical procedure is identical to that in the TAC group, with the exception of the ligation of the transverse aorta.

**Figure 1:**
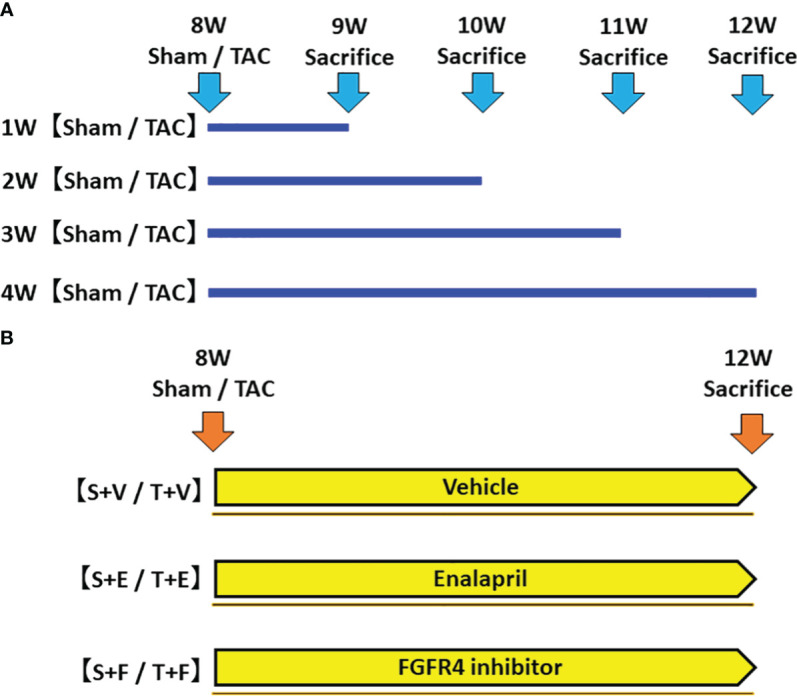
Protocol of the experiments. **(A)** The first experiment: Evaluation of changes in FGF23 levels and RAAS parameters during the development of LVH. We created a LVH model by TAC and evaluated these parameters at 1, 2, 3, and 4 weeks post-operation. **(B)** The second experiment: Effects of FGF23 and RAAS blockade on LVH. We created a LVH model by TAC and administered ACEi or FGFR4i post-operation. The animals were sacrificed, and their samples were evaluated 4 weeks after the operation and treatment. FGF23, fibroblast growth factor 23; RAAS, renin-angiotensin-aldosterone system; LVH, left ventricular hypertrophy; TAC, transverse aortic constriction; ACEi, angiotensin converting enzyme inhibitor; FGFR4i, fibroblast growth factor receptor 4 inhibitor.

In the first experiment, to investigate the time-course changes in FGF23 levels, RAAS parameters, and other parameters, mice were sacrificed, and their samples were evaluated at 1, 2, 3, and 4 weeks after sham operation (Sham group) or TAC operation (TAC group) (**Figure 1A**).

In the second experiment, mice were randomly assigned to the following six groups: (i) sham-operated mice treated with vehicle (S + V group, n = 5); (ii) sham-operated mice treated with enalapril maleate (S + E group, n = 6); (iii) sham-operated mice treated with H3B-6527, which is a highly selective fibroblast growth factor receptor 4 inhibitor (FGFR4i) (S + F, n = 6); (iv) TAC mice treated with vehicle (T + V group, n = 5); (v) TAC mice treated with enalapril maleate (T + E group, n = 6); and (vi) TAC mice treated with H3B-6527 (T + F group, n = 6). The treatment with vehicle, enalapril maleate, or H3B-6527 was started at postoperative day 1 and continued until sacrifice at 12 weeks of age (**Figure 1B**). Enalapril maleate was dissolved in drinking water at a concentration of 0.15 g/L and administered at liberty in the drinking water (estimated dose of 10-15 mg/kg body weight). The dose was decided by referring to previous studies (18, 19). H3B-6527 was prepared by dissolving stock solution (3 mM) in dimethyl sulfoxide (DMSO) and then diluting using phosphate-buffered saline. It was administered intraperitoneally daily (estimated dose of 10 mg/kg body weight). The dose was decided by referring to a previous study (20). H3B-6527 is a covalent inhibitor that targets the distinctive hinge cysteine in the FGFR4 kinase domain, selectively inhibiting FGFR4 while sparing other FGFRs. This compound plays a blocking role (21). Vehicle treatment involved daily intraperitoneal injections of DMSO.

All animal procedures were approved by the Institutional Animal Care and Use Committee at Kobe University Graduate School of Medicine (permit number: P210618) and were in strict accordance with the recommendations stipulated by the Guide for the Care and Use of Laboratory Animals of the National Institutes of Health. All efforts were made to minimize suffering, and the evaluations were reported in accordance with the ARRIVE guidelines (22).

### Biochemical analysis

Urine was collected from each mouse, held in an individual metabolic cage (Tecniplast, Exton, PA, USA), over a 24-h period. Blood samples were collected from the left ventricle before sacrificing under anesthesia. Serum samples were centrifuged for 5 minutes at 860 G and were stored at −80°C until analysis. Urine samples were also stored at −80°C for subsequent analysis.

Serum urea nitrogen and creatinine levels were measured using a Fuji Dri-Chem 3500 system (FUJIFILM Japan, Tokyo, Japan). Serum calcium and phosphorus levels were measured using Calcium E-Test Wako and Phospha C-Test Wako (FUJIFILM Wako Pure Chemical Industries, Osaka, Japan), respectively. Serum intact parathyroid hormone (iPTH), intact fibroblast growth factor 23 (iFGF23), and aldosterone levels were measured using ELISA (enzyme-linked immunosorbent assay) kits (mouse PTH 1-84 ELISA kit: Immutopics International, San Clemente, CA, USA; FGF23 ELISA kit: KAINOS laboratories, Inc., Tokyo, Japan; Aldosterone ELISA kit: Abcam, Cambridge, UK).

### Blood pressure measurement

Systolic blood pressure was measured by tail-cuff plethysmography (MK-2000; Muromachi Kikai Co., Ltd., Tokyo, Japan). To reduce the possibility of stress artifacts, mice were allowed to acclimatize to the environment for at least 15 minutes, and the mean of 10 measurements was then calculated.

### Echocardiographic measurements

Mice were mildly anesthetized with 1.5%-2% isoflurane mixed with 0.5-1.0 L/min 100% O_2_, and then, echocardiography was performed using a commercially available echocardiography system (F37; Hitachi Aloka Medical, Ltd., Tokyo, Japan). A two-dimensional short-axis view of the left ventricle was obtained at the papillary muscle level.

### Histological analysis

A transverse section of the heart was embedded in paraffin, sectioned, and stained with hematoxylin and eosin for histological analysis. Cross-sectional areas or diameters of myocardial fibers were analyzed using image analysis software (LUMINA VISION version 3.7.4.2; Mitani Corp., Tokyo, Japan). Immunohistochemical staining was performed for angiotensin-converting enzyme (ACE), angiotensin-converting enzyme 2 (ACE2), angiotensin II, and FGF23 using anti-ACE antibody (Abcam), anti-ACE2 antibody (R&D Systems, Minneapolis, MN, USA), anti-angiotensin II antibody (Novus Biologicals, Littleton, CO, USA), and anti-FGF23 antibody (R&D Systems). Areas with positive immunohistochemical staining areas were analyzed using image analysis software (LUMINA VISION version 3.7.4.2).

### RNA extraction and real−time polymerase chain reaction

Obtained heart samples were stored at -80°C until later analysis. For bone samples, the right femur was extracted, and the proximal metaphysis was isolated from the diaphysis. After flushing the bone with phosphate-buffered saline to eliminate the bone marrow, it was promptly frozen in liquid nitrogen. Subsequently, the right proximal metaphysis and diaphysis of the femur, devoid of bone marrow, were finely powdered using a mortar and pestle under liquid nitrogen in RNase-free conditions. Total RNA was extracted from the samples using RNAiso Plus (Takara Bio Inc., Shiga, Japan) and a high-salt solution for precipitation for bone (Takara Bio Inc., Shiga, Japan). The extracted RNA serves as the template for cDNA synthesis with the ReveTra ACE qPCR RT kit (TOYOBO Co., Ltd., Osaka, Japan) according to the manufacturer’s instructions. Real-time polymerase chain reaction (RT-PCR) was performed on the ABI 7500 system (Thermo Fisher Scientific, Waltham, MA, USA) using the SYBR Green Assay with Thunderbird SYBR qPCR Mix (TOYOBO). We analyzed the results using the 7500 System SDS Software Version 2.0.1 (Thermo Fisher Scientific). The mRNA expression levels of target genes were normalized to the expression level of glyceraldehyde-3-phosphate dehydrogenase (GAPDH). The primer sequences used in this study were as follows: atrial natriuretic peptide (ANP): forward 5’-AGGCAGTCGATTCTGCTTGA, reverse 5’-CGTGATAGATGAAGGCAGGAAG; brain natriuretic peptide (BNP): forward 5’-TAGCCAGTCTCCAGAGCAATTC, reverse 5’-TTGGTCCTTCAAGAGCTGTCTC; FGF23: forward 5’-ACAAGGACACCTAAACCGAACAC, reverse 5’-AGCTACTGACTGGTCCTATCACAGAA; angiotensinogen (AGT): forward 5’-TCTCTTTACCCCTGCCCTCT, reverse 5’-GAAACCTCTCATCGTTCCTTG; ACE: forward 5’-CCCTAGAGAAAATCGCCTTCTTG, reverse 5’-CGAAGATACCACCAGTCGAAGTT; ACE2: forward 5’-TGGGCAAACTCTATGCTG, reverse 5’-TTCATTGGCTCCGTTTCTTA; angiotensin II type 1 receptor (AT1R): forward 5’-CCATTGTCCACCCGATGAAG, reverse 5’-TGCAGGTGACTTTGGCCAC; GAPDH: forward 5’- GCAAAGTGGAGATTGTTGCCA, reverse 5’- AATTTGCCGTGAGTGGAGTCA.

### Statistical analysis

Values are presented as mean ± standard deviation (SD). Differences in the data between the corresponding T and S groups were analyzed using the Student’s t-test. Differences in the data among the groups treated with vehicle, enalapril maleate, or H3B-6527 were analyzed using one-way analysis of variance followed by the Holm method. Pearson’s correlation coefficient or Spearman’s rank correlation coefficient was used to analyze relationships between variables as appropriate. A two-tailed *p-*value of <0.05 was considered statistically significant. Statistical analyses were performed using IBM SPSS Statistics version 24.0 (IBM Corp., Armonk, NY, USA).

## Results

### Changes in animal characteristics and biochemical data during LVH progression

**Figure 2:**
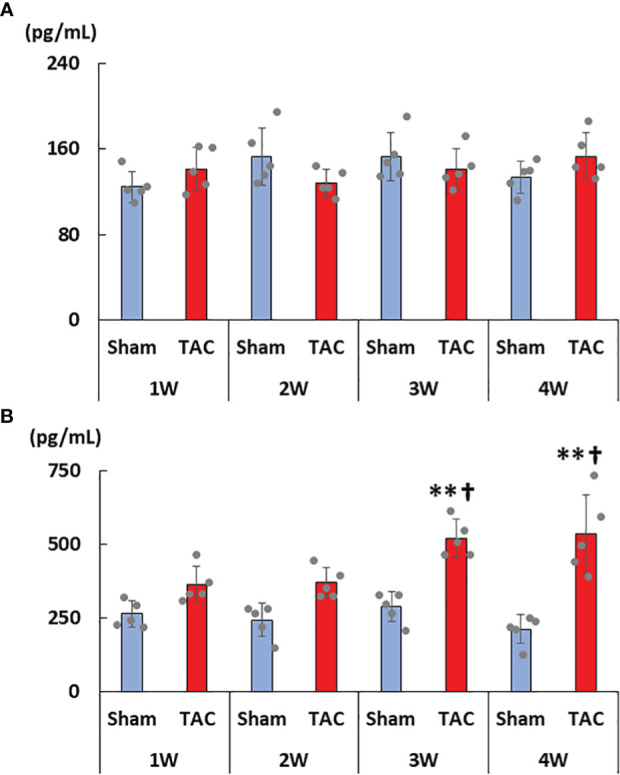
Changes in serum FGF23 and aldosterone levels. **(A)** Serum intact FGF23 levels. **(B)** Serum aldosterone levels. FGF23, fibroblast growth factor 23; TAC, transverse aortic constriction. Values are expressed as means ± SD. The blue bars represent values in the sham group and the red bars represent values in the TAC group at 1, 2, 3, and 4 weeks post-operation. * *p* < 0.05 and ** *p* < 0.01 versus the corresponding group. † *p* < 0.05 and ‡ *p* < 0.01 versus TAC at 1 week.

As for the first study, the characteristics and biochemical data of the mice at sacrifice are shown in **Table 1**. Heart weight increased in a time-dependent manner and was greater in the TAC group than in the Sham group. There were no significant differences in systolic blood pressure, and serum creatinine, calcium, and phosphorus levels at all weeks. Serum aldosterone levels were significantly higher in the TAC group than in the Sham group; however, serum iFGF23 levels did not change in both group during the study period (**Figures 2A, B**). As expected, echocardiographic findings showed that LVH gradually progressed in the TAC mice (**Table 2**).

**Table 1:** Animal characteristics at each week after TAC in experiment 1.

	1 wk		2 wk		3 wk		4 wk	
	Sham (n = 5)	TAC (n = 5)	Sham (n = 5)	TAC (n = 5)	Sham (n = 5)	TAC (n = 5)	Sham (n = 5)	TAC (n = 5)
BW (g)	24.3 ± 0.5	24.2 ± 1.2	26.1 ± 2.0	25.6 ± 1.1	24.8 ± 1.0	26.1 ± 0.7	25.6 ± 1.5	26.4 ± 1.8
HW (mg)	98.1 ± 4.7	117.4 ± 11.2^a^	103 ± 6.9	136.9 ± 6.1^be^	98.4 ± 5.8	160.8 ± 13.4^ce^	101.7 ± 5.5	178.3 ± 17.8^de^
RHW (g/100g BW)	0.41 ± 0.02	0.49 ± 0.03^a^	0.40 ± 0.01	0.54 ± 0.04^be^	0.40 ± 0.01	0.62 ± 0.04^ce^	0.40 ± 0.01	0.68 ± 0.05^de^
SBP (mmHg)	93 ± 2	96 ± 4	93 ± 6	92 ± 6	94 ± 4	96 ± 6	93 ± 4	93 ± 8
HR (bpm)	471 ± 95	464 ± 44	513 ± 69	431 ± 31	426 ± 54	450 ± 34	521 ± 60	456 ± 51
sCr (mg/dL)	0.30 ± 0.07	0.22 ± 0.08	0.34 ± 0.11	0.26 ± 0.05	0.32 ± 0.11	0.28 ± 0.04	0.26 ± 0.09	0.20 ± 0.07
sCa (mg/dL)	8.34 ± 0.89	8.03 ± 0.36	7.95 ± 0.55	8.54 ± 0.62	7.92 ± 0.49	7.08 ± 0.71	7.91 ± 0.6	7.78 ± 1.06
sP (mg/dL)	6.92 ± 0.79	7.34 ± 0.35	6.77 ± 1.16	7.76 ± 0.5	7.62 ± 0.72	7.4 ± 0.91	7.17 ± 0.76	7.71 ± 1.14

^a^ p < 0.05 versus sham group at 1 week; ^b^ p < 0.05 versus sham group at 2 weeks; ^c^ p < 0.05 versus sham group at 3 weeks; ^d^ p < 0.05 versus sham group at 4 weeks; ^e^ p < 0.05 versus TAC group at 1 week.

TAC, transverse aortic constriction; BW, body weight; HW, heart weight; RHW, relative heart weight; SBP, systolic blood pressure; HR, heart rate; sCr, serum creatinine level; sCa, serum calcium level; sP, serum phosphate level.

**Table 2:** Echocardiographic findings at each week after TAC in experiment 1.

	1 wk		2 wk		3 wk		4 wk	
	Sham (n = 5)	TAC (n = 5)	Sham (n = 5)	TAC (n = 5)	Sham (n = 5)	TAC (n = 5)	Sham (n = 5)	TAC (n = 5)
EF (%)	63.4 ± 0.7	61.5 ± 1.7	64.5 ± 1.6	63.2 ± 4.3	62.7 ± 1.6	62 ± 2.8	63.4 ± 2.1	62.7 ± 2.5
FS (%)	29.4 ± 0.4	28.2 ± 1.1	30.2 ± 1.0	29.4 ± 2.8	29.0 ± 1.0	28.6 ± 1.8	29.5 ± 1.5	29.0 ± 1.6
LVM (mg)	89.8 ± 13.6	121.6 ± 13.2^a^	83.7 ± 12.1	133.5 ± 7.5^b^	87.2 ± 7.9	172.8 ± 11.9^cf^	86.5 ± 15.2	200.7 ± 36^df^
LVAW (mm)	0.83 ± 0.08	0.99 ± 0.06^a^	0.73 ± 0.04	1.07 ± 0.04^b^	0.76 ± 0.08	1.19 ± 0.01^cf^	0.73 ± 0.06	1.28 ± 0.12^df^
LVPW (mm)	0.87 ± 0.10	0.97 ± 0.06	0.72 ± 0.10	1.04 ± 0.03^b^	0.72 ± 0.10	1.10 ± 0.05^c^	0.73 ± 0.10	1.15 ± 0.08^df^
LVDd (mm)	3.21 ± 0.28	3.40 ± 0.20	3.52 ± 0.20	3.38 ± 0.18	3.56 ± 0.34	3.68 ± 0.20	3.57 ± 0.09	3.83 ± 0.40
LVDs (mm)	2.25 ± 0.20	2.44 ± 0.18	2.46 ± 0.17	2.40 ± 0.19	2.53 ± 0.26	2.63 ± 0.15	2.52 ± 0.06	2.71 ± 0.33

^a^ p < 0.05 versus sham group at 1 week; ^b^ p < 0.05 versus sham group at 2 weeks; ^c^ p < 0.05 versus sham group at 3 weeks; ^d^ p < 0.05 versus sham group at 4 weeks; ^e^ p < 0.05 versus TAC group at 1 week

AC, transverse aortic constriction; EF, ejection fraction; FS, functional shortening; LVM, left ventricular mass; LVAW, left ventricular anterior wall thickness; LVPW, left ventricular posterior wall thickness; LVDd, diastolic left ventricular diameter; LVDs, systolic left ventricular diameter.

### Changes in the cardiac expressions of FGF23 and RAAS- and LVH-related factors during LVH progression

**Figure 3:**
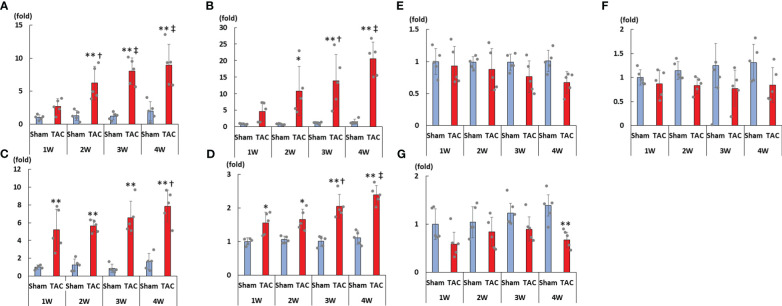
mRNA expressions of LVH-related factors in the heart tissue. **(A)** FGF23. **(B)** ANP. **(C)** BNP. **(D)** ACE. **(E)** AGT. **(F)** AT1R. **(G)** ACE2. LVH, left ventricular hypertrophy; FGF23, fibroblast growth factor 23; TAC, transverse aortic constriction; ANP, atrial natriuretic peptide; BNP, brain natriuretic peptide; ACE, angiotensin converting enzyme; AGT, angiotensinogen; AT1R, angiotensin II type 1 receptor; ACE2, angiotensin-converting enzyme 2. Values are expressed as means ± SD. The blue bars represent values in the sham group and the red bars represent values in the TAC group at 1, 2, 3, and 4 weeks post-operation. * *p* < 0.05 and ** *p* < 0.01 versus the corresponding group. † *p* < 0.05 and ‡ *p* < 0.01 versus TAC at 1 week.

**Figure 4:**
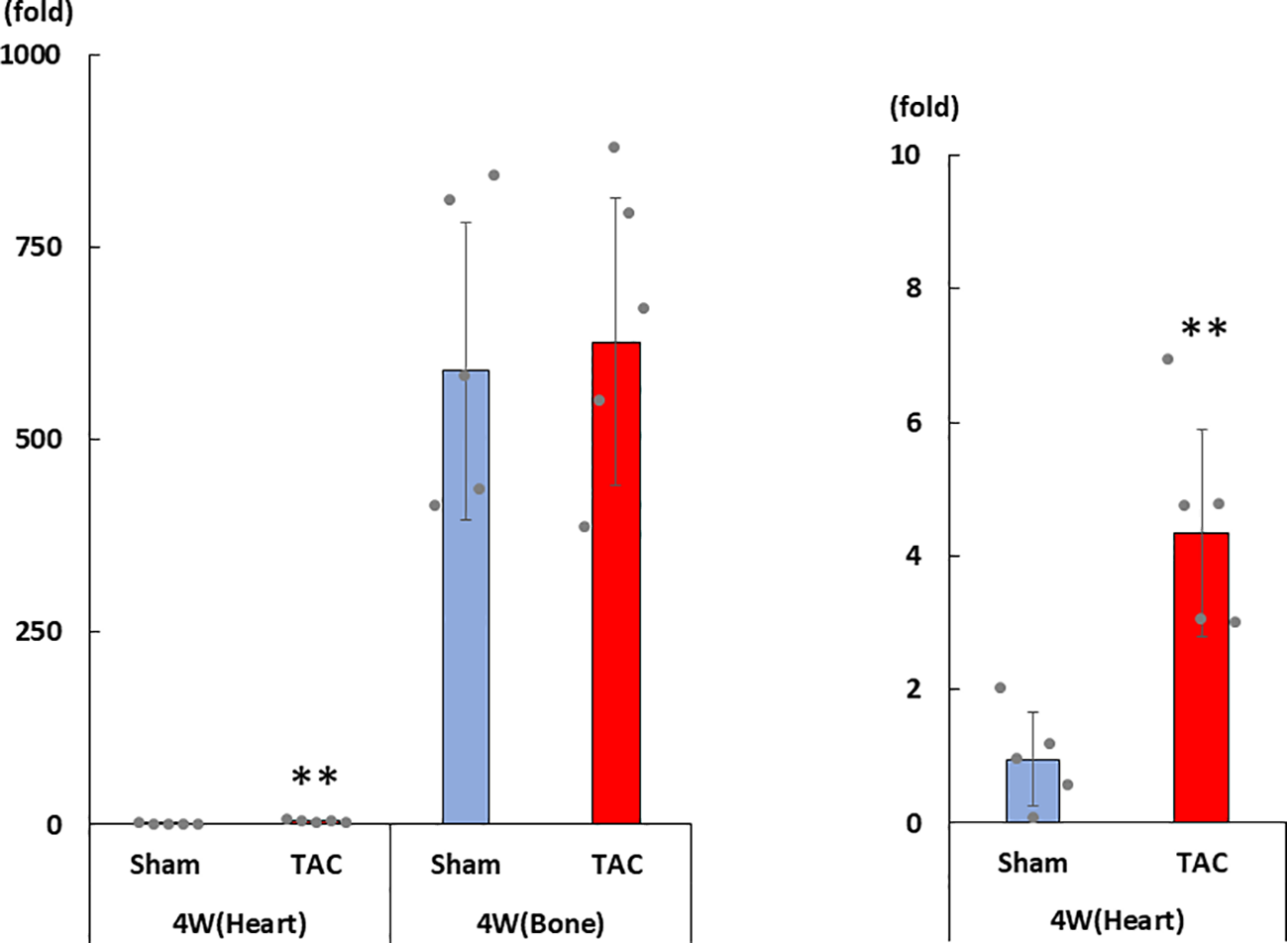
mRNA expression of FGF23 in the heart and the bone at 4 weeks after TAC. FGF23, fibroblast growth factor 23; TAC, transverse aortic constriction. Values are expressed as means ± SD. The blue bars represent values in the sham group and the red bars represent values in the TAC group at 1, 2, 3, and 4 weeks post-operation. * *p* < 0.05 and ** *p* < 0.01 versus the corresponding group.

As shown in **Figures 3A–D**, cardiac mRNA expressions of FGF23, ANP, BNP, and ACE gradually increased with the development of LVH. Although cardiac mRNA expressions of AGT and AT1R did not significantly differ between the study groups, the expression of ACE2 was significantly lower in the TAC group than in the Sham group at 4 weeks after surgery (**Figures 3E–G**). Furthermore, the mRNA expression of FGF23 was significantly and extremely lower in the heart than that in the bone at 4 weeks after TAC (**Figure 4**).

### Effects of RAAS and FGF23 inhibition on LVH

To examine the influence of the RAAS and FGF23 on LVH, we additionally studied the effect of blocking the RAAS and FGF receptor 4 (FGFR4) on LVH in TAC mice, using enalapril and H3B-6527, which is an FGFR4i.

At 12 weeks of age, the characteristics and biochemical data of the mice treated with vehicle, enalapril, or FGFR4i are shown in **Table 3**. Systolic blood pressure, and serum creatinine, calcium, phosphorus, iPTH, and iFGF23 levels did not significantly differ among the groups. Serum aldosterone levels were elevated in TAC mice and decreased on enalapril treatment. Conversely, FGFR4i treatment did not decrease the levels (**Table 3**).

**Table 3:** Animal characteristics after each treatment for 4 weeks in experiment 2.

	S + V (n = 5)	S + E (n = 5)	S + F (n = 5)	T + V (n = 5)	T + E (n = 5)	T + F (n = 5)
BW (g)	25.8 ± 1.6	26.2 ± 1.1	25 ± 0.8	25.3 ± 1.8	26.1 ± 1.6	25.5 ± 1.3
HW (mg)	102 ± 10	97 ± 6	99 ± 2	142 ± 15	119 ± 10**†	142 ± 18
RHW (g/100g BW)	0.39 ± 0.02	0.37 ± 0.01	0.40 ± 0.01	0.56 ± 0.05**	0.46 ± 0.03**†	0.56 ± 0.06**
SBP (mmHg)	96 ± 11	92 ± 8	93 ± 3	87 ± 7	89 ± 10	85 ± 9
HR (bpm)	542 ± 31	542 ± 31	551 ± 35	495 ± 50	519 ± 66	535 ± 33
sCr (mg/dL)	0.22 ± 0.06	0.17 ± 0.04	0.2 ± 0.03	0.23 ± 0.06	0.19 ± 0.04	0.19 ± 0.04
sCa (mg/dL)	7.7 ± 0.3	7.2 ± 1.1	7.6 ± 0.2	7.7 ± 0.5	8.2 ± 0.4	7.7 ± 0.1
sP (mg/dL)	8.3 ± 0.5	9.2 ± 1.1	9.1 ± 0.7	9.1 ± 0.7	9.8 ± 0.9	9.1 ± 0.7
iFGF23 (pg/mL)	111.1 ± 17.3	106.8 ± 14.2	149.7 ± 37.9	110.5 ± 24.1	104.8 ± 7.9	163.5 ± 49.2
Ald (pg/mL)	205.9 ± 30.4	224.5 ± 83.7	168.8 ± 33.8	396.4 ± 84.9**	158.8 ± 35.9‡	340 ± 53.6**

* p < 0.05 versus S + V group; ** p < 0.01 versus S + V group; † p < 0.05 vs T + V group; ‡ p < 0.01 versus T + V group.

BW, body weight; HW, heart weight; RHW, relative heart weight; SBP, systolic blood pressure; HR, heart rate; sCr, serum creatinine level; sCa, serum calcium level, sP, serum phosphorus level; iFGF23, serum intact fibroblast growth factor 23 level; Ald, serum aldosterone level.

**Figure 5:**
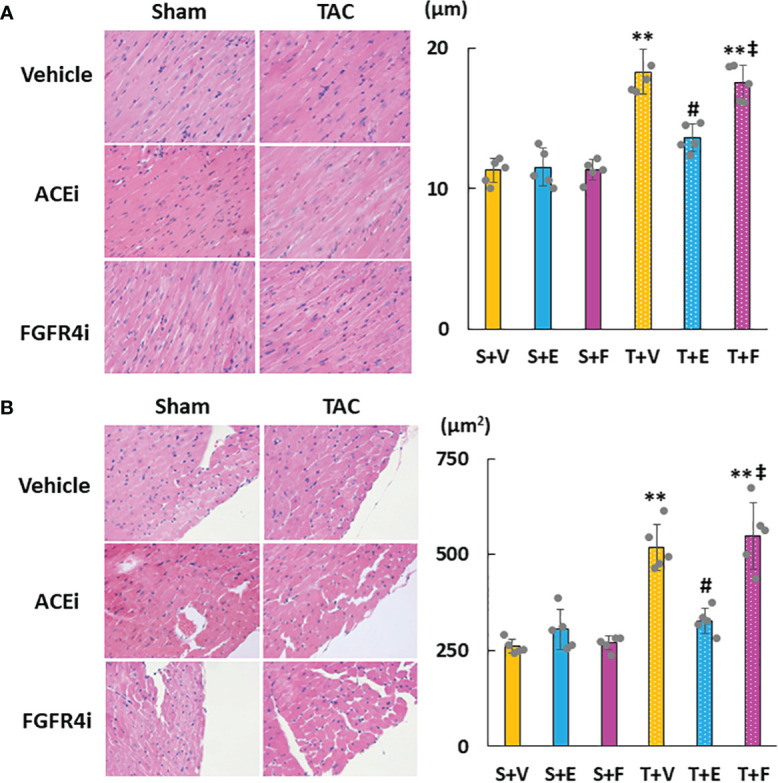
Evaluation of cardiac morphology. **(A)** Diameter of myocardial fiber. **(B)** Cross-sectional area of cardiomyocytes. Values are expressed as means ± SD. A yellow solid bar represents a value in the sham + vehicle (S+V) group, a blue solid bar represents values in the sham + enalapril (S+E) group, and a pink solid bar represents values in the sham + FGFR4i (S+F) group. A yellow dotted bar represents values in the TAC + vehicle (T+V) group, a blue dotted bar represents values in the TAC + enalapril (T+E) group, and a pink dotted bar represents values in the TAC + FGFR4i (T+F) group at 4 weeks after operation and following treatment. * *p* < 0.05 and ** *p* < 0.01. † *p* < 0.05 and ‡ *p* < 0.01 versus S + V. # *p* < 0.05 and ## *p* < 0.01 versus T + V.

Heart weight gain was suppressed in TAC mice treated with enalapril; however, there was no change in TAC mice treated with FGFR4i. On echocardiography, LVH in TAC mice was ameliorated by enalapril treatment but not FGFR4i treatment (**Table 4**). Histological analysis indicated that the cardiomyocyte width and area decreased with enalapril treatment but not with FGFR4i treatment (**Figure 5**).

**Table 4:** Echocardiographic findings after each treatment for 4 weeks in experiment 2.

	S + V (n = 5)	S + E (n = 5)	S + F (n = 5)	T + V (n = 5)	T + E (n = 5)	T + F (n = 5)
EF (%)	65.0 ± 1.2	65.5 ± 1.9	65.4 ± 0.8	64.2 ± 1.6	65.1 ± 1.0	63.4 ± 1.1
FS (%)	30.5 ± 0.9	30.8 ± 1.3	30.8 ± 0.5	29.9 ± 1.1	29.5 ± 2.8	29.3 ± 0.7
LVM (mg)	88.4 ± 18.2	75.8 ± 12.7	80.7 ± 11.9	147.2 ± 21.0**	112.9 ± 18.0†	137 ± 10.7**
LVAW (mm)	0.82 ± 0.09	0.75 ± 0.04	0.76 ± 0.09	1.29 ± 0.1**	1.07 ± 0.08†	1.22 ± 0.08**
LVPW (mm)	0.7 ± 0.05	0.65 ± 0.05	0.65 ± 0.05	1.07 ± 0.07**	0.9 ± 0.07‡	1.05 ± 0.05**
LVDd (mm)	2.42 ± 0.21	2.36 ± 0.19	2.47 ± 0.06	2.29 ± 0.28	2.24 ± 0.16	2.2 ± 0.11
LVDs (mm)	3.48 ± 0.28	3.4 ± 0.24	3.52 ± 0.09	3.2 ± 0.29	3.22 ± 0.27	3.18 ± 0.11
EF (%)	65.0 ± 1.2	65.5 ± 1.9	65.4 ± 0.8	64.2 ± 1.6	65.1 ± 1.0	63.4 ± 1.1

* p < 0.05 versus S + V group; ** p < 0.01 versus S + V group; † p < 0.05 vs T + V group; ‡ p < 0.01 versus T + V group

TAC, transverse aortic constriction; EF, ejection fraction; FS, functional shortening; LVM, left ventricular mass; LVAW, left ventricular anterior wall thickness; LVPW, left ventricular posterior wall thickness; LVDd, diastolic left ventricular diameter; LVDs, systolic left ventricular diameter.

**Figure 6:**
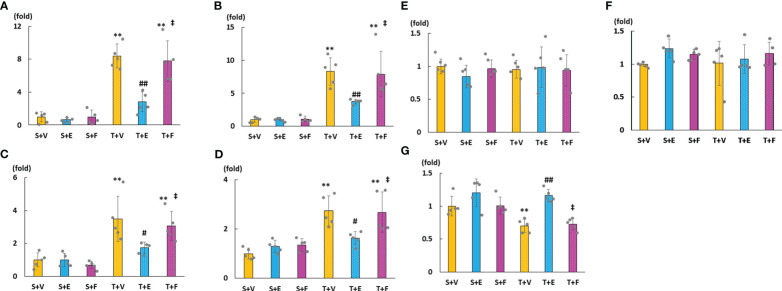
mRNA expressions of LVH-related factors in heart tissue. **(A)** FGF23. **(B)** ANP. **(C)** BNP. **(D)** ACE. **(E)** AGT. **(F)** AT1R. **(G)** ACE2. FGF23, fibroblast growth factor 23; ANP, atrial natriuretic peptide; BNP, brain natriuretic peptide; ACE, angiotensin converting enzyme; AGT, angiotensinogen; AT1R, angiotensin II type 1 receptor; ACE2, angiotensin-converting enzyme 2. Values are expressed as means ± SD. A yellow solid bar represents values in the sham + vehicle (S+V) group, a blue solid bar represents values in the sham + enalapril (S+E) group, and a pink solid bar represents values in the sham + FGFR4i (S+F) group. A yellow dotted bar represents values in the TAC + vehicle (T+V) group, a blue doted bar represents values in the TAC + enalapril (T+E) group, and a pink dotted bar represents values in the TAC + FGFR4i (T+F) group at 4 weeks after operation and following treatment. * *p* < 0.05 and ** *p* < 0.01 versus the corresponding group. † *p* < 0.05 and ‡ *p* < 0.01 versus S + V. # *p* < 0.05 and ## *p* < 0.01 versus T + V.

**Figure 7:**
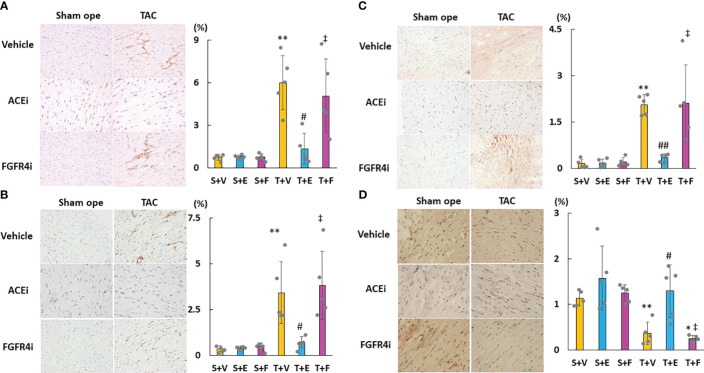
Evaluation of the protein expressions of LVH-related factors in heart tissue using immunohistochemistry. **(A)** FGF23. **(B)** ACE. **(C)** Angiotensin II. **(D)** ACE2. LVH, left ventricular hypertrophy; FGF23, fibroblast growth factor 23; ACE, angiotensin-converting enzyme; AT1R, angiotensin II type 1 receptor; ACE2, angiotensin-converting enzyme 2. Values are expressed as means ± SD. A yellow solid bar represents values in the sham + vehicle (S+V) group, a blue solid bar represents values in the sham + enalapril (S+E) group, and a pink solid bar represents values in the sham + FGFR4i (S+F) group. A yellow dotted bar represents values in the TAC + vehicle (T+V) group, a blue dotted bar represents values in the TAC + enalapril (T+E) group, and a pink dotted bar represents values in the TAC + FGFR4i (T+F) group at 4 weeks after operation and following treatment. * *p* < 0.05 and ** *p* < 0.01 versus the corresponding group. † *p* < 0.05 and ‡ *p* < 0.01 versus S + V. # *p* < 0.05 and ## *p* < 0.01 versus T + V.

As shown in **Figures 6A–D**, cardiac mRNA expressions of FGF23, ANP, BNP, and ACE increased in the T + V and T + F groups but decreased in the T + E group. Although the mRNA expressions of AGT and AT1R did not significantly differ among the groups, the mRNA expression of cardiac ACE2 was significantly higher in the T + E group than in the T + V and T + F groups (**Figures 6E–G**). Immunohistochemical staining analysis revealed that FGF23, ACE, and angiotensin II protein expressions increased in the T + V group and decreased in the T + E group (**Figures 7A–C**). In addition, the expression of ACE2 decreased in the T + V group and increased in the T + E group (**Figure 7D**). However, these expressions in the T + F group were similar to those in the T + V group (**Figures 7A–D**).

## Discussion

The present study demonstrated that in the early stage of LVH without CKD, (i) not serum FGF23 but cardiac FGF23 levels significantly increased during LVH progression; (ii) cardiac FGF23 expression was lower in the heart than in the bone in the TAC group; (iii) serum aldosterone levels and cardiac ACE expression significantly increased and cardiac ACE2 expression significantly decreased in the TAC group; and (iv) ACEi could prevent LVH progression but FGF4Ri could not.

In previous experimental studies, it was reported that the RAAS induced FGF23 production. *In vitro* studies showed that aldosterone and angiotensin II upregulated the expression of FGF23 in osteoblastic cells and neonatal rat ventricular myocytes and mineralocorticoid receptor blockers reversed this effect (23, 24). Moreover, an *in vivo* study showed that FGF23 expression was significantly upregulated in mouse hearts in response to angiotensin II infusion (25). In the present study, serum FGF23 levels did not increase during LVH progression in the absence of CKD; however, serum aldosterone levels increased. By contrast, intracardiac FGF23 expression significantly increased. Our recent study also found that there was a significant relationship between the expression of intracardiac FGF23 and the expression of intracardiac RAAS-related factors (26). Uninephrectomy-induced RAAS activation was reported to cause AMP-activated protein kinase (AMPK) inhibition in the remnant kidney of rats (27). Moreover, the AMPK activator down-regulated FGF23 transcription and AMPKa1-knockout mice had high serum FGF23 levels (28). Considering these results, the RAAS seems to regulate FGF23 production. Conversely, previous studies reported that FGF23 increased ATII production in neonatal rat ventricular myocytes (22, 23). Additionally, ATII receptor antagonists significantly attenuated FGF23-induced cellular hypertrophy. Therefore, it is speculated that FGF23-mediated activation of the RAAS in the heart might promote LVH (29, 30). In FGF23 knockout mice, chronic FGF23 administration decreased the renal expression of the ace2 gene but short-term FGF23 administration did not (31). Our study showed that ACEi reduced intracardiac FGF23 expression and upregulated intracardiac ACE2 expression. Although these findings indicate that there are interactions between FGF23 and the RAAS, a causal relationship between them remains unclear. Considering that serum FGF23 levels did not increase during LVH progression and not FGFR4i but ACEi prevented RAAS activation in the present study, we speculate that local RAAS activation may induce the increase in cardiac FGF23 levels.

Previous studies reported that FGF23 was associated with LVH. In an experimental study in mice, direct injection of FGF23 into the heart or systemic administration of a high-dose recombinant FGF23 induced LVH (11). In animal models and patients with CKD, FGF23 was reported to induce LVH via FGFR4 activation without klotho (12, 32). As previous experimental studies were conducted using specific animal models such as kl/kl mice, FGFR4^–/–^ mice, and, knock-in mice with an FGFR4 gain-of-function mutation, it remains unclear whether FGF23 directly induces LVH in clinical settings. In contrast, other previous studies reported that FGF23 was not associated with LVH. Patients with FGF23-related hypophosphatemic diseases did not show LVH, and no significant correlations between LVH and serum FGF23 levels were observed (33, 34). Even in animal studies, X-linked hypophosphatemia model mice, which showed high serum FGF23 levels and hyperphosphatemia without kidney dysfunction, did not manifest LVH (15, 35). LVH was induced by TAC in mice with global genetic ablation of FGF23 (36) and neutralization of FGF23 by treatment with FGF23-Ab did not improve LVH in CKD rats fed a high-phosphate diet and resulted in increased serum phosphate levels and aortic calcification associated with an increased risk of mortality (14). Conditional knockout mice for osteoblast/osteocyte-specific disruption of the FGF23 gene with CKD induced by adenine showed more severe LVH, although the elevation of serum FGF23 levels was suppressed compared to that in mice with only CKD, and therefore, increased bone FGF23 is necessary rather than harmful to protect against the cardiorenal consequences of elevated tissue phosphate. Moreover, the development of LVH in CKD is multifactorial and not solely dependent on markedly elevated serum FGF23 levels (37). *In vitro* application of phosphate alone increased the expression of LVH-related markers (15) and treatment with phosphate binder improved hyperphosphatemia and LVH, although serum FGF23 levels remained higher in CKD mice (38). These findings suggest that a high serum FGF23 level alone does not always cause LVH, and serum phosphate levels are also important for LVH. The heart produced FGF23 in animal models of pathological conditions, including heart failure, LVH, and myocardial infarction (15, 39–41). In these studies, serum FGF23 levels were also elevated, probably due to kidney dysfunction followed by heart failure. The present study showed that not serum FGF23 but cardiac FGF23 expression increased and was significantly correlated with the RAAS in the early stage of LVH induced by overload pressure without obvious kidney dysfunction. Our study also revealed that not FGFR4i but ACEi ameliorated LVH and RAAS parameters. Taking these results into account, we speculate that the activation of RAAS might induce LVH along with an increase in cardiac FGF23 levels.

In TAC mice, previous studies reported that activation of the RAAS plays a key role in the LVH progression (42, 43). Similarly, in the present study, activated RAAS parameters were significantly correlated with LVH parameters (data not shown), and ACEi ameliorated LVH. Our previous study also reported that more severe LVH was induced in TAC mice than in CKD rats although serum FGF23 levels were comparable between the two groups (26). In CKD rats, suppression of the RAAS by the administration of a vitamin D receptor activator prevented LVH though serum FGF23 levels remained high (44). In the present study, serum aldosterone levels increased twofold in the TAC group although serum FGF23 levels did not significantly change. These results suggest that the main factor that contributed to LVH was the RAAS rather than FGF23 in TAC mice.

Our study has several limitations. First, we showed changes in FGF23 levels and RAAS parameters only in the early stage of LVH without CKD. If these variables were observed for a much longer time or in the presence of CKD, the findings might differ from the results of the present study. Second, although we inhibited the FGF23 signal using FGF4Ri in the present study, we could not examine the pathophysiological changes associated with the inhibition of the heart-specific FGF23 signal. Third, the influence of inhibiting or activating ACE2 on cardiac FGF23 remains unclear in our study. Fourth, utilizing the tail-cuff method for blood pressure measurement has the potential to induce stress-induced changes in blood pressure due to animal restraint, despite being a nonoperative and cost-effective alternative compared to the telemetry method. Further studies are needed in the near future to clarify these issues.

## Conclusions

We speculate that the RAAS activation may be more important than FGF23 signaling in the early stage of pressure-overload-induced LVH. A further study is needed to clarify how FGF23 and the RAAS interact with each other in detail and whether FGF23 contributes to LVH in various situations.

## Data availability statement

The original contributions presented in the study are included in the article/Supplementary Material. Further inquiries can be directed to the corresponding author.

## Ethics statement

All animal procedures were approved by the Institutional Animal Care and Use Committee at Kobe University School of Medicine (permit number: P160707-R1) and were in strict accordance with the recommendations stipulated by the Guide for the Care and Use of Laboratory Animals of the National Institutes of Health.

## Author contributions

HF: Conceptualization, Supervision, Writing – review & editing, Writing – original draft. KW: Investigation, Writing – original draft. KO: Investigation, Writing – review & editing. KK: Data curation, Project administration, Writing – review & editing. SG: Data curation, Formal analysis, Writing – review & editing. SN: Supervision, Writing – review & editing.
